# DAP10 Predicted the Outcome of Pediatric B-Cell Acute Lymphoblastic Leukemia and Was Associated with the T-Cell Exhaustion

**DOI:** 10.1155/2021/4824868

**Published:** 2021-11-25

**Authors:** Nana Shi, Yingwan Luo, Ying Xu, Junyu Liang, An Ma, Yichao Gan, Bowen Wu

**Affiliations:** ^1^Department of Hematology-oncology, Children's Hospital, Zhejiang University School of Medicine, National Clinical Research Center for Child Health, Hangzhou, China; ^2^Department of Hematology, The First Affiliated Hospital, Zhejiang University School of Medicine, Hangzhou, China; ^3^Department of Rheumatology, The First Affiliated Hospital, Zhejiang University School of Medicine, Hangzhou, China; ^4^School of Basic Medical Sciences and Forensic Medicine, Hangzhou Medical College, Hangzhou, China; ^5^Institute of Genetics, Zhejiang University and Department of Genetics, Zhejiang University School of Medicine, Hangzhou, China; ^6^Cancer Center, Zhejiang University, Hangzhou, China

## Abstract

B-cell acute lymphoblastic leukemia is the most common malignant tumor in children. About 10–15% of patients will relapse with a 5-year OS of 57.5% for the past 20 years. As tumor microenvironment plays an important role in the disease process, many types of immunotherapy are approached. New immunotherapies including CAR-T cells have been developed for refractory B-ALL treatment. However, CAR-T treatment faces several problems, including loss of the target antigen and in vivo T-cell persistence. Here, we analyzed the tumor microenvironment of pediatric B-ALL patients in TARGET database. Using Cox analysis and PPI network, we finally sorted out the DAP10 gene. We found that DAP10 was hardly expressed in leukemic B cells. DAP10 was downregulated in B-ALL compared with normal individuals, and low expression level of DAP10 predicted poor survival. Furthermore, we found the tumor microenvironment was different in DAP10 high and low expression children. The CD8+ T cells might be hard to activate and more likely to suffer from exhaustion in DAP10 lowly expressed children. In conclusion, our results showed that DAP10 was a well biomarker to indicate the prognosis and tumor microenvironment in pediatric B-ALL. The treatment strategy of immunotherapy for the leukemic children with DAP10 lowly expressed should be adjusted if needed.

## 1. Introduction

Acute lymphoblastic leukemia (ALL) is the most common malignant tumor in children characterized by the proliferation of immature lymphoid cells, and B-cell lineage (B-ALL) accounts for the majority. Over the past few decades, there have been significant improvements in the survival outcomes of pediatric ALL. Analyses showed the 5-year overall survival (OS) rate for children was from 86% to 90% [1, 2]. Despite these improvements, 10–15% of patients will relapse and approximately 55% of those will relapse again [3, 4]. In contrast to the improvement of survival in newly diagnosed patients, the outcome of relapsed patients remains poor with a 5-year OS of 57.5% and has not changed significantly for the past 20 years [5, 6].

Cancer cells are the initial cause of tumor development and progression. Tumor microenvironment (TME) plays a pivotal role in the process and it also affects therapeutic efficacy [7–9]. Many studies have demonstrated that patients with high immune infiltration would improve clinical outcomes and have better treatment response [10, 11]. As more research studies of TME have emerged, immunotherapeutic approaches increased. New immunotherapeutic strategies have been developed for refractory B-ALL treatment and achieved clinical success, including chimeric antigen receptor (CAR) T cells and monoclonal antibodies [12, 13]. Long-term remission has been reported in patients who received CAR-T cells [14]. Given the efficacy of CAR-T cells treatment from several clinical trials, Tisagenlecleucel was approved by the FDA in 2017 for treating patients 25 years or younger with relapsed or refractory B-ALL. However, CAR-T cells treatment faces several problems, including neurotoxicity, cytokine release syndrome, B-cell aplasia, loss of the target antigen, and in vivo T-cell persistence [15–17]. In addition, previous studies have shown that the overall survival of adult B-ALL patients progressing after CD19 CAR-T cells is poor. Longer remission duration from CAR-T cells was associated with superior survival after progression following CAR-T therapy [18]. Therefore, longer remission time after CAR-T therapy should be considered.

Here, we analyzed the tumor microenvironment of pediatric B-ALL patients in TARGET database and eventually sorted out the DAP10 gene. We found that DAP10 was downregulated in B-ALL compared with normal individuals, and low expression level of DAP10 predicted poor survival. Furthermore, we found the tumor microenvironment was different in DAP10 high and low expression children. The CD8+ T cells might be hard to be activated and more likely to suffer from exhaustion in DAP10 lowly expressed children. Thus, the strategy of immunotherapy of these children should be adjusted if needed to achieve longer remission.

## 2. Materials and Methods

### 2.1. Ethics Statements

This study was approved by the Ethics Committee of the Children's Hospital, Zhejiang University School of Medicine (Zhejiang, China). Bone marrow samples from children with acute B-cell lymphoblastic leukemia were obtained from Children's Hospital, Zhejiang University School of Medicine, with their informed consent in accordance with the Declaration of Helsinki.

### 2.2. Data Selection

The search strategy used for TCGA was provided in Table S1. In such case, 440 cases were firstly obtained. Then, the data were further purified by the conditions in Table S1 in turn. There were 97 patients involved in our analysis. Both FPKM and Count data were downloaded.

### 2.3. Analysis of Tumor Microenvironment (TME)

The tumor microenvironment was analyzed by ESTIMATE [19] package and single sample gene set enrichment analysis (ssGSEA) [20] in R software. Feature gene panels for each immune cell type in ssGSEA were obtained from a recent publication [21].

### 2.4. Analysis of Differentially Expressed Genes (DEGs)

For the data of TARGET database, the DEGs were calculated with the DESeq2 package in R software. The DEGs of the high immune score group compared with the low immune score group and the high stromal group compared with the low stromal score group were analyzed, respectively. |log_2_ (fold change)| more than 1 and *P* value less than 0.05 were set as cutoffs to pick up the DEGs. Then, an intersection was used to identify the common genes shared by the immune score group and stromal score group.

For the data of GEO database, we took average expression for genes with duplicates before analysis. Then, the DEGs were calculated with the limma package in R software. |log_2_ (fold change)| more than 1 and *P* value less than 0.05 were set as cutoffs to pick up the DEGs if needed.

### 2.5. Analysis of Gene Ontology (GO) and Kyoto Encyclopedia of Genes and Genomes (KEGG)

The GO and the KEGG analysis were done using the package in R software.

### 2.6. Analysis of Protein-Protein Interaction (PPI) Network

The PPI network was analyzed in String database and reconstructed with Cytoscape version 3.8.0.

### 2.7. Gene Set Enrichment Analysis (GSEA)

Hallmark and C7 collection version 7.1 were downloaded from Molecular Signatures Database. The data were analyzed with GSEA software version 4.0.2. The gene sets with NOM *P* value less than 0.05 and FDR Q value less than 0.25 were considered to be enriched in the analysis.

### 2.8. Western Blot

Samples, obtained from bone marrow cells of pediatric ALL and peripheral blood of healthy donors, were lysed with 10 times of volume of red blood cell lysate and then washed with PBS for twice. Cells were collected and lysed in protein extraction reagent (Thermo Fisher Scientific) containing protease and phosphatase inhibitor (Thermo Fisher Scientific). The proteins were separated in SDS-page and transferred to PVDF membranes (Bio-Rad). The DAP10 antibody was from Hua-Bio (ER1910-39) and the GAPDH antibody was from Thermo Fisher Scientific. The bound antibodies were visualized using SuperSignal reagents (Thermo Fisher Scientific).

### 2.9. Flow Cytometry (FCM)

For analysis of surface markers, cells were stained in PBS containing 2% BSA. The following fluorescent-labeled antibodies were used: DAP10-Alexa Fluor 700 (R&D System), CD45-V500 (BD Biosciences), and CD19-APC (BD Biosciences). Flow cytometry data were collected using Canto-II (BD Biosciences) cytometers and analyzed with Flowjo V10.0.7 software.

### 2.10. Statistical Analysis

The Kaplan–Meier survival and Cox model were done in R software, as well as the analysis of DEGs and the correlations. For general data, the statistical analysis was performed in SPSS software version 21.0. Student's t-test and the nonparametric test were applied for quantitative data according to their normality. The chi-square test was used for the qualitative data. Statistically significant differences were indicated as follows: ^*∗*^ for *P* < 0.05, ^*∗∗*^ for *P* < 0.01, and ^*∗∗∗*^ for *P* < 0.001.

## 3. Results

### 3.1. Strategy of Data Selection

The transcriptional data were obtained from the TCGA database. We chose TARGET program and added all the data from projects of ALL-P1, ALL-P2, and ALL-P3. The disease type was limited in the lymphoid leukemia, and the sample type was the primary blood derived cancer bone marrow. The details of search strategy were listed in Table S1. 441 cases were available after the survey. The strategy of purifying the data was in Table S1. We removed the cases of T-ALL, mixed-ALL, no survival data, or those above 18 years old according to the clinical data. The cases merely containing transcriptional data of blood sample or relapsed bone marrow sample were not enrolled in the analysis. At last, 97 cases of pediatric B-ALL samples were involved in the subsequent work.

### 3.2. Tumor Microenvironment Was Associated with Survival and Relapse in Pediatric B-ALL

The tumor purity and scores of tumor microenvironment were calculated by the ESTIMATE package in R. Immune score (IS) indicated the proportion of immune cells, whereas stromal score (SS) showed the ratio of stromal cells in tumor tissue. ESTIMATE score (ES) was a comprehensive indicator of these two scores.

**Figure 1:**
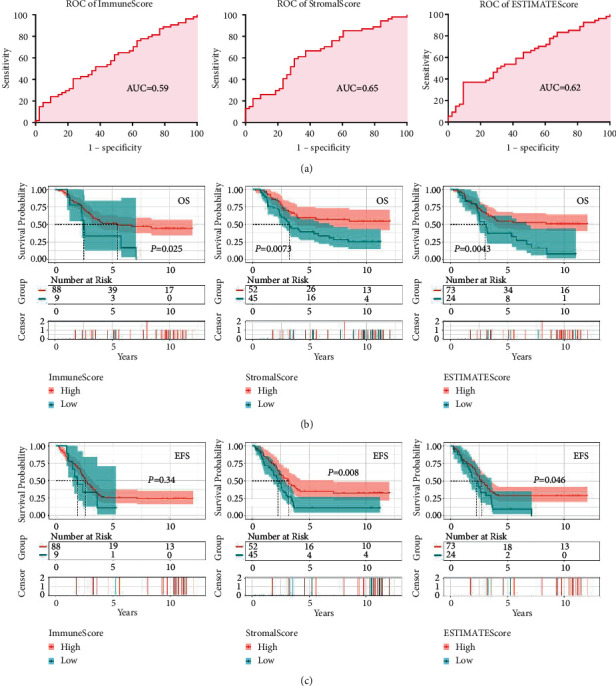
Tumor microenvironment (TME) was associated with survival in childhood B-ALL. (a) ROC curves of immune score, stromal score, and ESTIMATEscore. (b) High immune score, stromal score, and ESTIMATEscore predicted favorable overall survival. (c) High stromal score and ESTIMATEscore predicted favorable event free survival.

After each sample was scored, respectively, the scores were analyzed with the survival using K-M curve. As Figure 1, S1 showed, all the three scores were associated with survival in childhood B-ALL. Patients with high IS might suggest a longer OS than those with low IS (median OS, 5.5 versus 2.5 years, *P*=0.025, Figure 1(b)). Patients who had high SS (*P*=0.0073) or high ES (*P*=0.0043) had better OS than those who had low scores as well (Figure 1(b)). Using the same cutoffs, we then explored the relationships between the scores and the event free survival (EFS) or the relapse free survival (RFS). The results showed that high SS indicated favorable EFS and RFS (Figure 1(c), S1B). High ESTIMATE score was only correlated with favorable EFS (Figure 1(c)).

Next, we analyzed the correlations of TME with clinical features. As shown in Table 1, the relapse ratio in low SS group was significantly higher than that in high SS group (82.2% versus 63.5%, *P*=0.033). In addition, we found that patients who had a low stromal score were more likely to come along with TCF3-PBX1 fusion (17.6% versus 2.4%, *P*=0.028). There was no significant difference in the features of age, count of white blood cells (WBC), the third degree of central nervous system (CNS3), BCR-ABL1 status, TEL/AML1 fusion, trisomy 4/10, or the risk stratification between high score group and low score group. These results indicated that TME predicted the outcomes in pediatric B-ALL.

### 3.3. Identification of the Immune-Related Hub Genes Shared by the Immune Score Group and the Stromal Score Group

**Figure 2:**
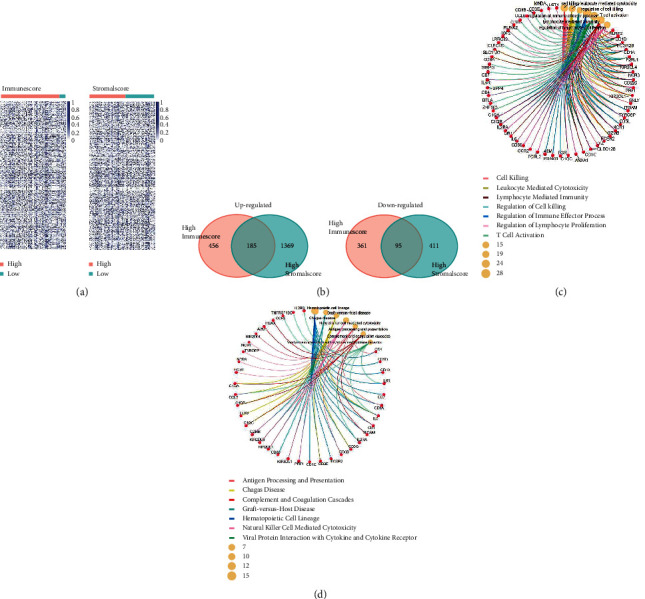
Hub genes took part in the tumor microenvironment in pediatric B-ALL. (a) DEGs of the high scores versus low scores shown by heatmaps. (b) Selection of the upregulated genes and downregulated genes shared by the high immune score groups and high stromal score groups. GO (c) and KEGG (d) analysis showed that the hub genes participated in the T-cell activation and lymphocyte mediated immunity.

Since both high immune score and high stromal score indicated better survival, it was worth exploring the differential expressed genes (DEGs) in the two groups. As the results showed (Figures 2(a) and 2(b)), there were 641 genes upregulated and 456 genes downregulated in the IS group (high versus low scores). In the SS group, the numbers of upregulated genes and downregulated genes were 1554 and 506, respectively (high versus low scores). After intersections, a total of 185 hub upregulated genes and 95 hub downregulated genes were found among the two groups.

**Figure 3:**
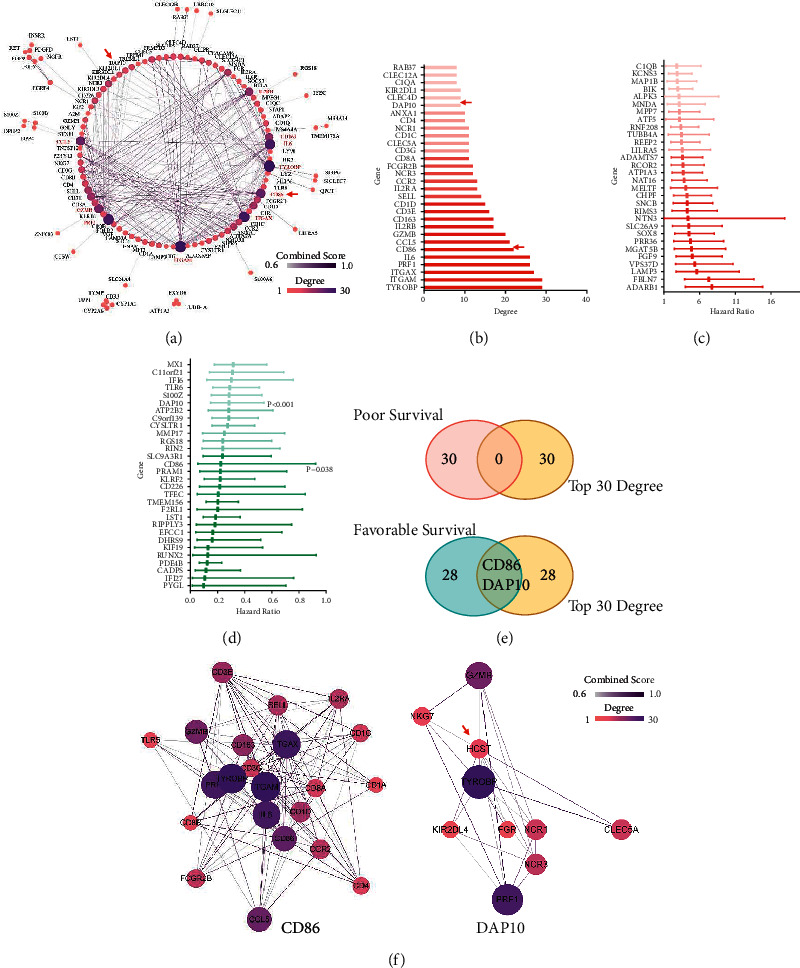
The gene DAP10 was identified after the PPI and Cox screening process. (a) Protein-protein interaction network of the hub genes. (b) Top 30 genes ranked by the degree of nodes in the PPI network. Top 30 genes of favorable (c) and poor (d) genes of survival ranked by HR after Cox analysis. (e) Intersections of the top genes in PPI network with the top genes in COX analysis. (f) PPI network of CD86 and DAP10 (HCST).

To further explore the functions of these hub genes, we performed GO and KEGG analysis. As Figure 2(c) showed, these hub genes mainly participated in the biological process of cell killing, leukocyte mediated cytotoxicity, lymphocyte mediated immunity, regulation of cell killing, regulation of immune effector process, regulation of lymphocyte proliferation, and T-cell activation. In accordance with the GO analysis, the results of KEGG also showed that these hub genes were involved in the pathways of antigen processing and presentation, natural killer cell mediated cytotoxicity, Th17 cell differentiation, and hematopoietic cell lineage (Figure 2(d)). We then constructed the protein-protein interaction (PPI) to show the network of the hub genes (Figure 3(a)). The top 30 genes ranked by degrees of nodes were listed in Figure 3(b). These might be the central genes in the PPI network and played critical roles in pediatric B-ALL.

To evaluate the correlations of the DEGs with survival, we then performed Cox analysis. The results revealed that a total of 172 genes independently predicted the survival. Among them, 76 genes indicated poor survival, whereas the other 96 genes suggested favorable survival. Both the top 30 genes in each group ranked by HR with *P* < 0.05 were listed in Figures 3(c) and 3(d). These genes were then overlapped with the top 30 genes according to degree in the PPI network. As Figures 3(e) and 3(f) showed, we at last gained two genes, CD86 and DAP10. These two genes both had a great impact on survival and played an important role in the PPI network.

### 3.4. Low Expression Level of DAP10 Predicted the Poor Survival and Relapse in Childhood B-ALL

**Figure 4:**
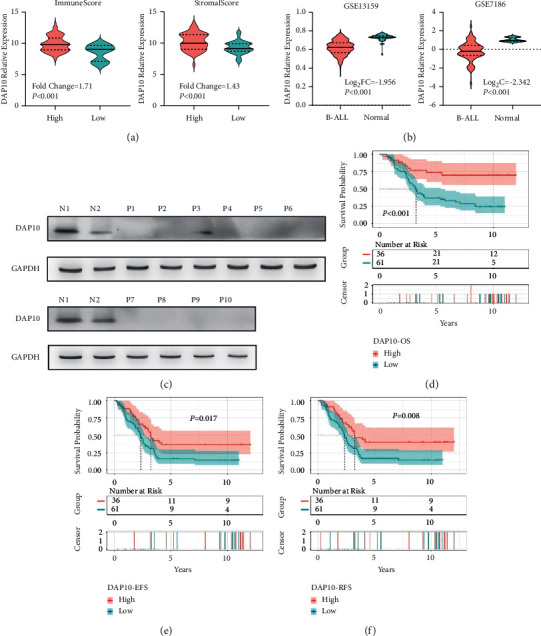
High expression level of DAP10 was associated with favorable survival. (a) Patients in high immune score and high stromal score groups showed higher expression level of DAP10. (b) Downregulation of DAP10 in B-ALL bone marrow compared to the normal individuals. (c) The results of western blot showed that DAP10 was hardly expressed in 10 pediatric ALL patients compared to 2 normal individuals. N: normal; P: patients. High expression level of DAP10 predicted favorable OS (d), EFS (e), and RFS (f).

As the function of CD86 had been well studied in B-ALL, we focused on DAP10 in our study. Compared with the low score groups, the mRNA expression levels of DAP10 were significantly upregulated in the high score groups (Figure 4(a)), with the fold changes of 1.71 (IS, *P* < 0.001) and 1.43 (SS, *P* < 0.001), respectively. The transcriptional data of normal bone marrow were unavailable in TCGA and TARGET database. So, we searched GEO database to demonstrate the DAP10 expression between patients and normal individuals instead. As Figure 4(b) showed, the expression of DAP10 decreased in B-ALL compared with those in the normal samples in two datasets, GSE7186 and GSE13159. The results of western blot showed that DAP10 was hardly expressed in pediatric ALL patients in protein level (Figure 4(c), Table S2). More importantly, patients with low expression level of DAP10 had worse OS, EFS, and RFS (Figures 4(d)–4(f), Table S3). We also analyzed the correlation between the DAP10 expression and the clinical features. As Table 2 demonstrated, the relapse ratio was significantly higher in the low DAP10 group compared to the high DAP10 group. Low expression level of DAP10 was associated with TCF3-PBX1 fusion as well.

### 3.5. Downregulation of DAP10 Inhibited the Activation of CD8+ T Cells in Pediatric B-ALL

**Figure 5:**
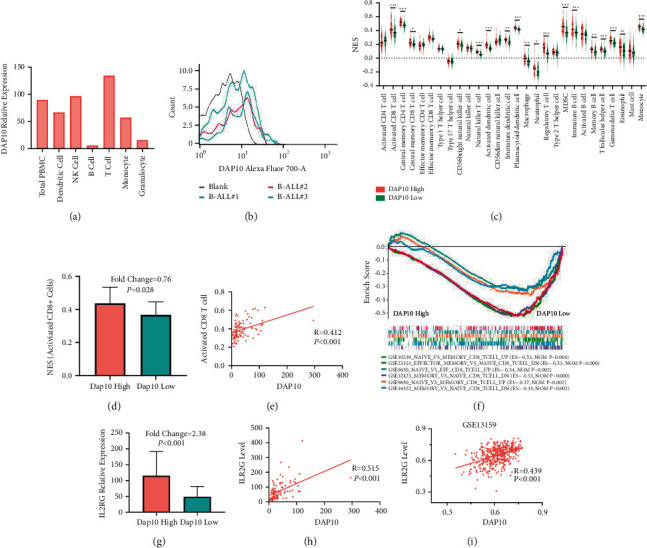
Downregulation of DAP10 inhibited the activation of CD8+ T cells. (a) mRNA level of DAP10 in different hematopoietic cells. (b) Fluorescence intensity of DAP10 of leukemic B cells using FCM. (c) ssGSEA showed the proportions of TICs in DAP10 high and DAP10 low patients. (d) The ratio of activated CD8+ T cells decreased in DAP10 low patients. (e) The ratio of CD8+ T cells was positively correlated with DAP10. (f) GSEA analysis showed DAP10 participated in the activation of CD8+ T cells. (g) The level of IL2RG was inhibited in the DAP10 low patients. (h), (i) The level of IL2RG was positively correlated with the level of DAP10 in the patients of TARGET database and GSE13159 dataset.

According to the database of Protein Atlas, DAP10 was mainly expressed on the T cells and NK cells but hardly expressed on the B cells (Figure 5(a)). We next used flow cytometry to study the expression of DAP10 on leukemic B cells. As shown in Figure 5(b), S2, the proportion of DAP10 positive cells and overall average fluorescence intensity in the leukemic B cells were hardly changed, compared to the blank, suggesting leukemic B cells hardly expressed DAP10. Therefore, the expression level of DAP10 had almost no relationship with leukemic B cells. Thus, DAP10 could be an ideal biomarker to indicate the tumor microenvironment of the bone marrow in pediatric B-ALL patients.

We used ssGSEA to study the differences of the TME between the patients in low and high DAP10 expression group. There were 18 tumor-infiltrating immune cell types downregulated in the DAP10 low expression group among all the 28 cell types (Figure 5(c)). We noticed that the expression level of activated CD8+ T-cell decreased in these children (Figure 5(d)), indicating that the cell-meditated cytotoxicity was inhibited in the bone marrow. The correlation study showed that the level of activated CD8+ T cells was positively associated with the expression level of DAP10 in all the 97 patients (*R* = 0.421, *P* < 0.001, Figure 5(e)). Consistently, we found that the genes related to naïve CD8+ T cells (compared with the effector CD8+ T cells or memory DC8+ T cells) were enriched in patients with low DAP10 expression (Figure 5(f)).

After antigen stimulation, CD8+ T cells express IL2 receptor as well as autocrine IL2 to promote their proliferation and differentiation. Interestingly, our study showed that the expression levels of IL2RB and IL2RG were significantly suppressed in patients with DAP10 low expression, whereas the expression level of IL2 did not change (Figure 5(g), S3). In accordance, the correlation analysis suggested that the levels of IL2RG and IL2RB both positively correlated with the level of DAP10 in TARGET database and in GSE13159 (Figures 5(h) and 5(i), S3). Since IL2RB and IL2RG were crucial in the intracellular signaling during the activation process of CD8+ T cells, these results demonstrated that CD8+ T cells in these patients could not transmit downstream signals to the nucleus when exposing to antigen, which led to the blockade of the activation of CD8+ T cells.

### 3.6. Downregulation of DAP10 Was Associated with the Exhaustion of CD8+ T Cells

**Figure 6:**
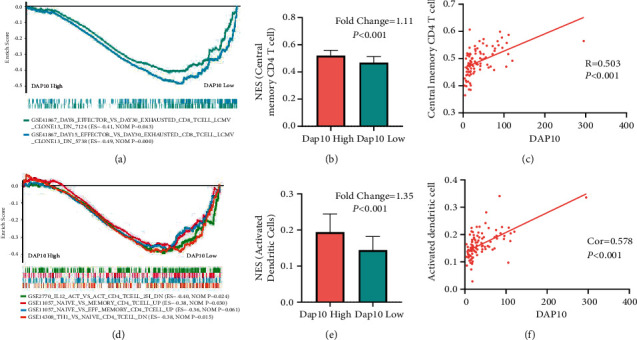
Cells that contributed to the T-cell exhaustion in the DAP10 lowly expressed patients. (a) GSEA analysis showed that patients with lower DAP10 level suffered from more severe T-cell exhaustion. (b) The ratio of central memory CD4+ T cells was downregulated in DAP10 low expression group. (c) The level of central memory CD4+ T cells was positively correlated with DAP10 expression. (d) The naive CD4+ T cells failed to activate and differentiate to type 1 CD4+ helper cells in DAP10 lowly expressed patients. (e) The ratio of activated dendritic cells was downregulated in DAP10 lowly expressed children. (f) The level of activated dendritic cells was positively correlated with DAP10 expression.

We also noticed that the genes related to CD8+ T-cell exhaustion were enriched in the DAP10 low expression group (Figure 6(a)). Consistently, the central memory CD8+ T cells decreased in those patients (Figure 5(c)). One of the characteristics of T-cell exhaustion was that the differentiation from the effector cells into memory cells was blocked. Thus, these results suggested that CD8+ T cells might be exhausted in patients with low DAP10 expression.

Previous studies have reported that multiple factors participated in the CD8+ T-cell exhaustion. These reasons included the altered helper T cells differentiation, APC dysfunction, increased expression of suppressive cytokines, and increased expression of inhibitory receptors [22]. In our analysis, the ratios of activated dendritic cells and central memory CD4+ T cells decreased in the DAP10 low expression group (Figures 6(b) and 6(e)). In consequence, the proportions of activated dendritic cells and the central memory CD4+ T cells showed positive correlation with the level of DAP10 (Figures 6(c) and 6(f)). Using GSEA, we found that the genes related to effector or memory CD4+ T cells were downregulated in the low DAP10 group. Moreover, genes that were enriched in the untreated CD4+ T cells group (compared with IL12 treated) were enriched in the DAP10 low patients, which might suggest that the CD4+ T cells could not differentiate with type 1 helper cells in these patients (Figure 6(d)).

**Figure 7:**
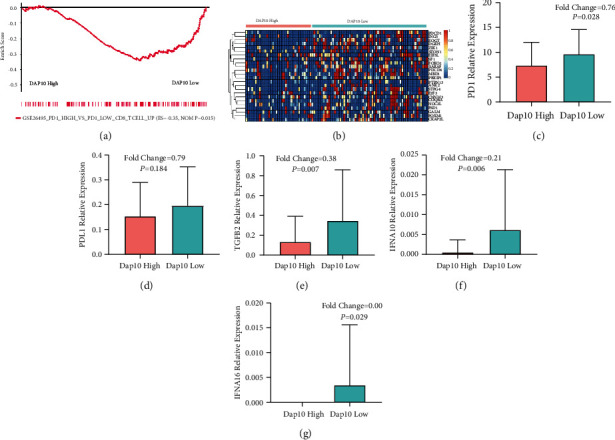
Molecules and pathways involved in the T-cell exhaustion in the DAP10 lowly expressed patients. (a) PD1 related pathway was upregulated in the DAP10 lowly expressed patients. (b) Heatmap showed that the genes related to PD1 were enriched in DAP10 low expression patients. (c) PD1 was upregulated in DAP10 low expression group. (d) PD-L1 had no significant correlation with DAP10 level. DAP10 lowly expressed patients showed increased suppressive cytokines of TGFB (e), IFNA10 (f), and IFNA16 (g).

**Figure 8:**
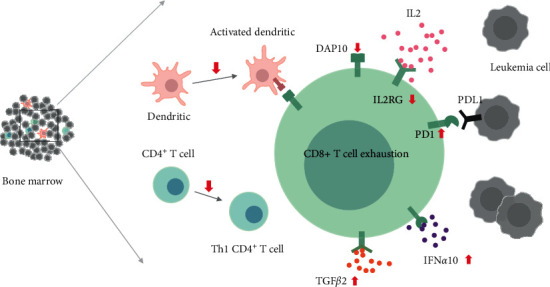
Cells and molecules involved in the CD8+ T-cell exhaustion in DAP10 low expression patients.

Interestingly, we also discovered that the genes associated with high expression level of PD1 were enriched in patients with DAP10 low expression (Figures 7(a) and 7(b)), which suggested that PD1 related pathways were upregulated in these patients. Furthermore, we found that PD1 was significantly highly expressed in these children as well (Figure 7(c)). However, there was no significant change of PD-L1 between the two groups (Figure 7(d)). We also found that TGFB2, IFNA10, and IFN16 were upregulated in these patients (Figure 7(e)–7(g)). These results above might suggest that the CD8+ T cells were exhausted in the children with DAP10 low expression (Figure 8).

## 4. Discussion

In the article, we studied the role of DAP10 in children with B-ALL. We found that the expression of DAP10 decreased in B-ALL compared to the normal individuals, and low expression of DAP10 indicated a poor prognosis and was associated with the relapse. Moreover, we found that the CD8+ T cells might fail to activate and more likely to be exhausted in DAP10 low expression patients.

The tumor microenvironment consists of immune cells, endothelial cells, mesenchymal cells, and extracellular matrix molecules and inflammatory mediators [23]. Immune and stromal cells, the two major cell types of nontumor components, have been reported to be valuable for predicting the prognosis of tumors [11, 24, 25]. We firstly calculated the scores of TME for 97 patients selected from the TARGET database by ESTIMATE package. The results showed that TME was associated with the prognosis in pediatric B-ALL. Then, we used the DESeq2 and screened out 280 differentially expressed genes. We found the DEGs were mainly involved in cytotoxicity from the GO and KEGG analyses, indicating our research strategy was reliable. To figure out the core genes, we analyzed the data using PPI networks and Cox analysis and finally identified the DAP10 gene. We then discovered the role of DAP10, revealing that DAP10 was downregulated in the pediatric B-ALL, and low expression level of DAP10 was correlated with poor prognosis. Moreover, low expression level of DAP10 was associated with relapse as well.

Furthermore, we found that the tumor microenvironment was different between DAP10 high expression and low expression groups. The differences were mainly in the activation and exhaustion of CD8+ T cells. We firstly used ssGSEA analysis and showed that the process of leukocyte mediated cytotoxicity was strongly inhibited in DAP10 low expression patients. In particular, the activation of CD8+ T cells was blocked in these children, which was in accordance with the previous studies that DAP10 acting as a costimulatory receptor of CD8+ T cells [26, 27]. Moreover, we found that IL2RG and IL2RB significantly decreased after DAP10 downregulation. Since these two proteins were crucial in the intracellular signaling during the activation process of CD8+ T cells [28, 29], we supposed that CD8+ T cells in DAP10 low expression patients could not transmit downstream signals to the nucleus when exposed to the antigen, which led to the blockade of activation of CD8+ T cells.

In addition, the CD8+ T cells suffered from exhaustion in DAP10 lowly expressed B-ALL children. Multiple factors contributed to the T-cell exhaustion [22]. In the aspect of cells, we found that the CD4+ T cells might fail to differentiate to the type 1 T helper cells. Moreover, the inhibition of the activation of dendritic cells might promote this process as well. The blockade of immune checkpoint was also involved. The PD1/PD-L1 pathway was upregulated in these patients leading to the programmed cell death of T cells. Besides, the cytokines such as TGF*β* or IFN*α* might also contribute to the exhaustion of T cells in these patients.

It was reported that 10–15% of the pediatric B-ALL patients would relapse. Our results found that the children with low expression level of DAP10 were more likely to relapse and the CD8+ T cells were hard to activate and suffered from the exhaustion. The immunotherapy could be selected as the subsequent therapies after disease relapse. Previous studies have shown that the overall survival of adult B-ALL patients progressing after CD19 CAR-T cells was poor. Longer remission duration from CAR-T cells was associated with superior survival after progression following CAR-T therapy [18]. Though the overall prognosis of pediatric B-ALL patients progressing after CAR-T therapy remained unknown, longer remission time after immunotherapy should be considered. When using CAR-T therapy, increasing the expression of DAP10 or adding an intracellular immunoreceptor tyrosine-based activation motif (ITAM) domain might improve the therapeutic effect in DAP10 low expression B-ALL children. The previous study had shown that when elongating two second-generation CD28/4-1BB/CD3*ζ* CARs by a DAP10 domain, these CAR-T cells showed superior antitumor activity against other cancers, such as gastric cancer, lung cancer, and hepatocellular carcinoma, in xenograft-bearing mice [30, 31]. The DAP10/CD3*ζ*/CD27 or the PD1-CD3*ζ*/DAP10 CAR-T cells performed better than the CD28/CD3*ζ* CAR-T cells as well [32–34]. In addition, antibodies of TGF*β* or IFN*α* could be chosen if the combination of immunotherapy was considered.

## 5. Conclusion

Our study showed that DAP10 was a well biomarker to indicate the prognosis and tumor microenvironment in pediatric B-ALL. The treatment strategy of immunotherapy for the leukemic children with DAP10 low expression should be adjusted if needed.

## Figures and Tables

**Table 1:** Clinical data of the groups divided by immune score and stromal score.

	Immune-H^a^	Immune-L	*P*	Stromal-H	Stromal-L	*P*
WBC count	53.1 ± 7.5	25.2 ± 9.7	0.419	56.2 ± 11.3	44.0 ± 7.2	0.779
WBC >50	36.4% (32/88)	11.1% (1/9)	0.121	38.5% (20/52)	33.3% (15/45)	0.378
Age	7.4 ± 5.0	7.9 ± 4.8	0.339	7.8 ± 5.1	7.4 ± 4.8	0.438
Age ≥10	33.0% (29/88)	33.3% (3/9)	0.623	32.7% (17/52)	33.3% (15/45)	0.559
BCR-ABL1	3.4% (3/88)	0.0% (0/9)	0.744	5.8% (3/52)	0.0% (0/45)	0.150
CNS3^b^	3.4% (3/88)	0.0% (0/9)	0.744	5.8% (3/52)	0.0% (0/45)	0.150
High risk	58.0% (51/88)	44.4% (4/9)	0.332	61.5% (32/52)	51.1% (23/45)	0.204
Relapse	71.6% (63/88)	77.8% (7/9)	0.518	63.5% (33/52)	82.2% (37/45)	0.033
Tri 4/10	12.8% (10/78)	0.0% (0/9)	0.315	10.9% (5/46)	12.2% (5/41)	0.554
TEL/AML1	11.6% (8/69)	11.1% (1/9)	0.723	11.1% (4/36)	11.9% (5/42)	0.599
TCF3/PBX1	9.0% (6/67)	11.1% (1/9)	0.602	2.4% (1/42)	17.6% (6/34)	0.028

For quantitative data, the results were presented as mean ± SEM; for qualitative data, the results were presented as percentage (positive/all). (a) Immune-H, high immune score group; Immune-L, low immune score group; Stromal-H, high stromal score group; Stromal-L, low stromal score group. (b) CNS3, CNS3 at diagnosis.

**Table 2:** Clinical data of the two groups divided by the RNA level of DAP10.

	DAP10 high	DAP10 low	*P*
WBC count	62.4 ± 13.9	43.5 ± 7.2	0.232
WBC >50	41.7% (15/36)	31.1% (19/61)	0.294
Age	7.3 ± 0.8	7.6 ± 0.6	0.753
Age ≥10	30.6% (11/36)	34.4% (21/61)	0.695
BCR-ABL1	2.8% (1/36)	3.3% (2/61)	1.000
CNS3 at diagnosis	8.3% (3/36)	0.0% (0/61)	0.048
High risk	58.3% (21/36)	60.7% (34/61)	0.803
Relapse	55.6% (20/36)	82.0% (50/61)	0.005
Tri 4/10	21.2% (7/33)	5.6% (3/54)	0.038
TEL/AML1	11.1% (3/27)	11.8% (6/51)	1.000
TCF3/PBX1	0.0% (0/27)	14.3% (7/49)	0.046

For quantitative data, the results were presented as mean ± SEM; for qualitative data, the results were presented as percentage (positive/all).

## Data Availability

The RNA-Seq data of 97 patients in our analysis were from the TCGA database.

## Supplementary Materials

Supplementary Materials Supplementary Table 1: search strategy of TCGA database and purification of data. Supplementary Table 2: clinical features of 10 pediatric ALL patients. Supplementary Table 3: multivariate analysis of DAP10 expression in survival. Supplementary Figure 1: high stromal score predicted favorable relapse free survival. Supplementary Figure 2: DAP10 was hardly expressed in leukemic B cells. Supplementary Figure 3: the level of IL2RB was decreased in the DAP10 low expression children.
