# Supplementary material for: DAP10 Predicted the Outcome of Pediatric B-Cell Acute Lymphoblastic Leukemia and Was Associated with the T-Cell Exhaustion
Source: J Oncol. 2021 Nov 25;2021:4824868. doi: 10.1155/2021/4824868 (PMC8639274; doi:10.1155/2021/4824868)
Supplement: Supplementary Materials — Supplementary Table 1: search strategy of TCGA database and purification of data. Supplementary Table 2: clinical features of 10 pediatric ALL patients. Supplementary Table 3: multivariate analysis of DAP10 expression in survival. Supplementary Figure 1: high stromal score predicted favorable relapse free survival. Supplementary Figure 2: DAP10 was hardly expressed in leukemic B cells. Supplementary Figure 3: the level of IL2RB was decreased in the DAP10 low expression children. [file 4824868.f1.docx]

**Supplementary Tables**

**Table S1. Search strategy of TCGA database and purification of data**

| Item | Results |
| --- | --- |
| Data Selection |  |
| Exploration |  |
| Primary Site | hematopoietic and reticuloendothelial systems |
| Program | TARGET |
| Project | TARGET-ALL-P1, TARGET-ALL-P2, TARGET-ALL-P3 |
| Disease Type | Lymphoid leukemias |
| Experimental Strategy | RNA-Seq |
| Sample Type | Primary blood derived cancer-bone marrow |
| Repository |  |
| Data Category | Transcriptome profiling |
| Data Type | Gene Expression Quantification |
| Experimental Strategy | RNA-Seq |
| Workflow Type | HTSeq-Counts, HTSeq-FPKM |
|  |  |
| Data Purification In Turn |  |
| T-ALL and Mix phenotype ALL | Removed 253 cases (T-ALL 245 cases, Mix 8 cases) |
| Unknown survival status | Removed 5 cases |
| Age > 18y | Removed 7cases |
| Blood sample only | Removed 34 cases |
| Recurrent blood derived cancer | Removed 15 cases |
| 09B Samples | Removed 29 cases |
|  | 97 cases remained |

**Table S2. Clinical features of 10 pediatric ALL patients**

| No. | Age (year) | Sex | WBC count (10^9^/L) | Bone marrow lymphoblast | Cytogenetics |
| --- | --- | --- | --- | --- | --- |
| 1 | 0.6 | Female | 142.2 | 95% | 45,X,add(X)(p21),-11[13]/46,idem,+18[2]/46,XX[5] |
| 2 | 2.7 | Male | 1.2 | 75% | 57,XY,+X,+4,+6,+7,+8,+10,+13,+17,+18,+21,+21[4]/46,XY[16] |
| 3 | 2.3 | Female | 53.2 | 84% | 46,XX[10]/46,XX,t(12;21)(p13;q22)[10] |
| 4 | 7.3 | Female | 6.4 | 71% | 46,XX,dup(1)(q21q42),dup3(q21q27),del(6)(q13q23),t(9,22)(q34;q11.2),t(14;15)(q32;q22)[20] |
| 5 | 6.6 | Female | 9.5 | 95% | 46,XX[20] |
| 6 | 7.3 | Female | 31.7 | 98% | 46,XX[20] |
| 7 | 1.5 | Female | 6.3 | 97% | 46,XX[4]/55,XX,+4,+4,+6,+9,+14,+17,+18,+21,+21[16] |
| 8 | 12.8 | Female | 56.1 | 96% | 46,XX[20] |
| 9 | 1.8 | Male | 5.6 | 24% | 62,XY,dup(1)(q21q32),+4,+5,+6,+7,+7,+8,+10,+11,+12,+13,+14,+14,+19,+21,+21,+22[4]/46,XY[16] |
| 10 | 13.7 | Male | 2.9 | 71% | 46,XX[20] |

**Table S3. Multivariate analysis of DAP10 expression in survival**

|  | P (OS) | OR1 (95%CI) | P (EFS) | OR2 (95%CI) |
| --- | --- | --- | --- | --- |
| DAP10 high | 0.001 | 0.299 (0.150-0.596) | 0.005 | 0.472 (0.280-0.793) |
| WBC>50 | 0.582 | 1.198 (0.630-2.280) | 0.071 | 1.717 (0.954-3.088) |
| Age≥10 | 0.141 | 0.635 (0.371-1.151) | 0.682 | 0.899 (0.540-1.497) |
| High Risk | 0.440 | 1.258 (0.703-2.251) | 0.270 | 1.335 (0.799-2.232) |

**Supplementary Figures**


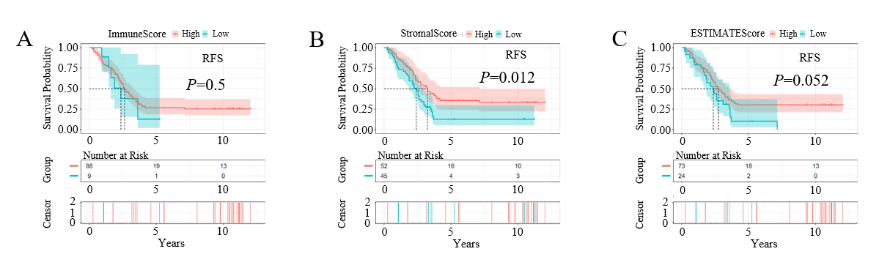


**Figure S1. High stromalscore predicted favorable relapse-free survival**


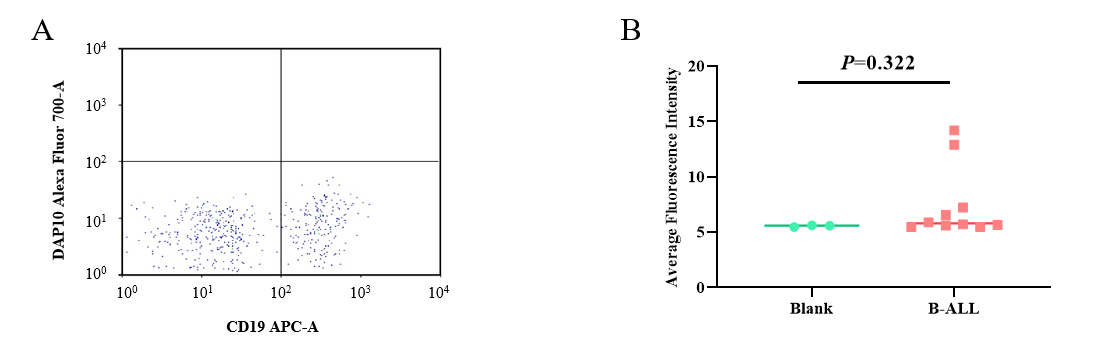


**Figure S2. DAP10 was hardly expressed in leukemic B cells.** (A) The results of FCM showed that leukemic B cells did not express DAP10. (B) There was no significance between leukemic B cells samples and blank in average fluorescence intensity.


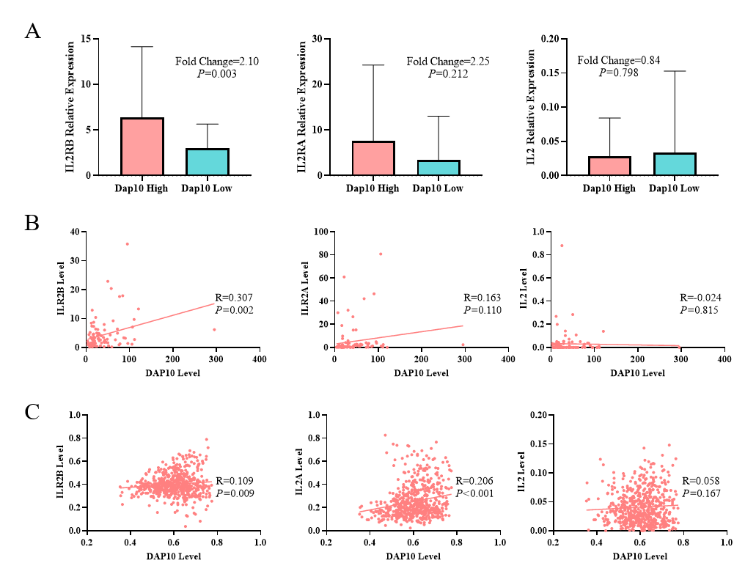


**Figure S3. The level of IL2RB was decreased in the DAP10 low expression children.** (A) The level of IL2RB, IL2RA and IL2 between DAP10 high expression and low expression groups. (B) Correlations of IL2RB, IL2RA and IL2 with DAP10 in patients of TARGET database. (C) Correlations of IL2RB, IL2RA and IL2 with DAP10 in children of GSE13159 dataset.
